# Protective Effects of Total Flavones of* Elaeagnus rhamnoides* (L.) A. Nelson against Vascular Endothelial Injury in Blood Stasis Model Rats

**DOI:** 10.1155/2017/8142562

**Published:** 2017-10-31

**Authors:** Zhicheng Wei, Fang Zuo, Wenqian Wang, Li Wang, Dong Tong, Yong Zeng, Ping Wang, Xianli Meng, Yi Zhang

**Affiliations:** ^1^Department of Pharmacology, School of Pharmacy, Chengdu University of Traditional Chinese Medicine, Chengdu 611137, China; ^2^College of Ethnic Medicine, Chengdu University of Traditional Chinese Medicine, Chengdu 611137, Sichuan Province, China

## Abstract

The aim was to evaluate the protective effects of total flavones of* Elaeagnus rhamnoides *(L.) A. Nelson (TFE) against vascular endothelial injury in blood stasis model rats and explore the potential mechanisms preliminarily. The model of blood stasis rat model with vascular endothelial injury was induced by subcutaneous injection of adrenaline combined with ice-water bath. Whole blood viscosity (WBV), histological examination, and prothrombin time (PT), activated partial thromboplastin time (APTT), and fibrinogen (FIB) were measured. Meanwhile, the levels of Thromboxane B2 (TXB_2_), 6-keto-PGF_1*α*_, von Willebrand factor (vWF), and thrombomodulin (TM) were detected. In addition, Quantitative Real-Time PCR (qPCR) was performed to identify PI3K, Erk2, Bcl-2, and caspase-3 gene expression. The results showed that TFE can relieve WBV, increase PT and APTT, and decrease FIB content obviously. Moreover, TFE might significantly downregulate the levels of TXB_2_, vWF, and TM in plasma and upregulate the level of 6-keto-PGF_1*α*_ in plasma. Expressions of PI3K and Bcl-2 were increased and the expression of caspase-3 was decreased by TFE pretreatment in the rat model. Consequently, the study suggested that TFE may have the potential against vascular endothelial injury in blood stasis model rats induced by a high dose of adrenaline with ice-water bath.

## 1. Introduction

Based on the guidance of traditional Chinese medicine theory, blood stasis syndrome (BSS) means blood stasis which mainly includes pain, lumps, bleeding, tongue purple, and pulse astringent in clinical [1]. Thrombosis is a multifactorial disease caused by a combination of blood stasis and hypercoagulable conditions [2]. It is generally known that thrombosis is closely related to activation of platelet adhesion, aggregation, secretion functions, and activation of intrinsic and extrinsic coagulation systems, which cause blood coagulation and fibrin formation [3]. Thus, BSS and thrombotic diseases are closely linked. Nowadays, thrombosis and its complications have become one of the world's highest rates of morbidity and mortality. However, the etiology and pathogenesis of thrombotic disease are very complex and has not been fully elucidated, for example, when the VEC morphology is injured or functional changes will hinder the normal operation of the blood, resulting in thrombosis [4, 5]. Thrombosis, especially triggered by vascular endothelial cell injury, is the pathological basis of many cardiovascular diseases [6, 7].

In recent years, with the development and in-depth study of Chinese medicine, many Chinese herbs and formulas have been found that they have the effects of benefitting blood transmission and eliminating blood stasis possessing obvious antiplatelet [8, 9]. Sea buckthorn (*Elaeagnus rhamnoides *(L.) A. Nelson), as a traditional Chinese and Tibetan medicine, has been used to treat various conditions. TFE, the major components of* Elaeagnus rhamnoides *(L.) A. Nelson, has been made as a marketed drug “Xin-Da-Kang Capsule” and used in the treatment of ischemic heart disease and ischemic cerebrovascular disease. During the past two decades, considerable works had been done to study and investigate the function of TFE in vascular disease. Here, a method for TFE has developed and reported that could inhibit in vitro platelet aggregation and attenuate vascular endothelial cell injury in vivo [10, 11]. By using network pharmacology methods, we had been found that TFE may be involved in the pathogenic mechanism of vascular endothelial cell injury to treat myocardial ischemia, and our results suggest that TFE may activate some genes associated with VECs apoptosis development, such as PI3K, Erk2, Bcl-2, and caspase-3 [12]. However, date is still less accurate, we need to provide more studies for the feasibility of the potential mechanisms. So the current studies were intended to evaluate protective effects of TFE in blood stasis model rats with vascular endothelial injury. We preliminarily focus on the effect of TFE on vascular endothelial cell apoptosis, and further studies will be summarized in our next work.

## 2. Materials and Methods

### 2.1. Reagents and Chemicals

Aspirin Enteric-coated Tablets (J20130078) was produced by Bayer Healthcare S.r.l. (Leverkusen, Germany) and dissolved in 0.5% CMC-Na to 3 mg/mL before using. Fufang Danshen Tablets (Z44022225) was bought from Guangdong Yili Group Pharmaceutical Holding Co., Ltd. (Sihui, China) and dissolved in 0.5% CMC-Na before using. Adrenaline Hydrochloride injection (H12020526) was manufactured by Tianjin Jinyao Pharmaceutical Co., Ltd. (Tianjin, China). activated partial thromboplastin time assay kit (F008-2), prothrombin time assay kit (F002), thrombin time assay kit (F009), fibrinogen (FIB) assay kit (F010), von Willebrand factor (vWF) ELISA kit (H274), and thrombomodulin (TM) ELISA kit (H273) were bought from Nanjing Jian Cheng Bioengineering Institute (Nanjing, China). TXB_2_ ELISA kit (ADI-900-002) and 6-keto-PGF_1*α*_ ELISA kit (ADI-900-004) were purchased from Enzo Life Sciences Inc. (New York, USA). Trizol reagent (15596026) was purchased from Invitrogen (Carlsbad, CA, USA). DEPC-water (PAB180005) was purchased from Bio-Swamp Life Science Lab (Wuhan, Hubei). DNase I (2270A) and reverse transcription kit (639505) were purchased from Takara Biomedical Technology (Beijing) Co., Ltd. (Beijing, China). SYBR green PCR kit (KM4101) was purchased from KAPA Biosystems (Boston, MA, USA). All of the other chemicals were of analytical-reagent grade.

### 2.2. Animals and Feeding Conditions

Adult male Sprague-Dawley (SD) rats (200 ± 20 g) were purchased from Sichuan Academy of Medical Sciences & Sichuan Provincial People's Hospital, the Institute of Experimental Animals (Chengdu, China). Animals were maintained at a temperature of 22–25°C and moisture of 55%–60%. All animals were allowed to eat and drink freely. This study was carried out in China State Administration of TCM, Chengdu University of TCM (TCM Pharmacology P3 laboratory, number TCM2032043). Experimental proceeding was rigorous in conformity with the Guide for the Care and Use of Laboratory Animals. The experimental design was approved by the Animal Ethics Committee of Chengdu University of Traditional Chinese Medicine.

### 2.3. Preparation of TFE

THE (PY20160902) provided by Nanjing PuYi Biological Technology Co., Ltd. (Nanjing, China). After drying, crushing, and supercritical degreasing, sea buckthorn pulp was extracted with 75% ethanol at 70°C for 2.5 h and repeated 3 times. The extract was separated by D101 macroporous resin and purified by silica gel column to collect the TFE. Finally, the TFE was dried under reduced pressure for about 30 hours to obtain the final product. TFE was dissolved in 0.5% CMC-Na and formulated into three different concentrations solution at 12, 24, and 48 mg/mL.

### 2.4. Standardization of TFE

In order to ensure the quality and precise administration of TFE, three main active compounds of total flavonoid aglycones were identified by high-performance liquid chromatography (HPLC). A ZORBAX Eclipse Plus-C8 LC column (3.0 × 150 mm, 1.8 *μ*m, Agilent, USA) was used with the column temperature sustained at 40°C. Methanol and 0.1% formic acid in water (48 : 52) were treated as mobile phases A and B, respectively. The flow rate was set at 0.6 mL/min, and the sample injection volume was set at 10 *μ*L. The detector wavelength was set at 370 nm. Under this chromatographic condition, quercetin, kaempferol, and isorhamnetin can achieve baseline separation and have good peak shape.

### 2.5. Model Establishment and Drug Administration

Rats were randomly parted into seven experimental groups (*n* = 6, per group). The control and model groups were given control solvent. Aspirin (ASP) group was given 0.03 g/kg ASP. Fufang Danshen Tablets (FFDSP) group was given 0.45 g/kg FFDSP. TFE administration (TFE-L, TFE-M, and TFE-H) groups were given 0.12, 0.24, and 0.48 g/kg TFE. All administration groups were administered by gavage, once a day for 15 days. From the 10th to 14th day, rats in the control group were injected subcutaneously with 0.9% (w/v) NaCl saline solution, and other rats subcutaneous subcutaneously injected diluted adrenaline hydrochloride (0.67 mg/mL, 0.5 mL/kg) at 10 am, 2 pm, and 6 pm daily. From the 13th to 14th days of the experiment, all rats were soaked in ice-cold water (0~4°C) for 5 min after each injection of adrenaline [13]. Before the last administration, the rats were fasted for 12 hours but were free to drink water.

### 2.6. Model Assessment

To evaluate the success of the model in rats, the hemorheology and histological examination were assessed in this study. According to a previously described method [14, 15], the hemorheology indexes of WBV and histological examination were measured. All rats were anesthetized by 20% urethane (0.5 ml/100 g, ip) after the last administration. Blood was collected from the abdominal aorta and placed in a plastic tube containing 3.8% sodium citrate (citrate/blood: 1/9, v/v). Plasma of each group was procured through centrifugation at 3000 rpm for 15 min. The WBV was determined by a blood rheology analyzer (SA-6000, Beijing Succeeder Technology Inc., China) at 1 s^−1^, 5 s^−1^, 50 s^−1^, 100 s^−1^, and 200 s^−1^ shear rates at 37°C. This experiment was completed within 3 h after blood collection.

### 2.7. The Coagulation Function Test

Blood was collected from the abdominal aorta and placed in a plastic tube containing 3.8% sodium citrate (citrate/blood: 1/9, v/v), and then plasma was separated through centrifugation of 3,000 rpm at 5°C for 15 min. PT, APTT, and FIB were measured by the Siemens Sysmex CA-500 Series analyzer and the kits according to instructions (Nanjing Jiancheng Bioengineering Institute, Nanjing, China). This experiment was completed within 3 h after blood collection.

### 2.8. Histological Examination

The thoracic aorta was fixed with 4% paraformaldehyde and embedded in paraffin. The paraffin blocks were then sectioned to a longitudinal section of 4 *μ*m thickness by microtome (RM2235, Leica Microsystems, Co., Ltd., Germany). Tissue sections were stained with hematoxylin and eosin (H&E), and histopathological changes were observed with the microscope (CX41, Olympus, Tokyo, Japan).

### 2.9. Determination of TXB_2_, 6-Keto-PGF_1*α*_, vWF, and TM in the Plasma

The blood sample was collected from the abdominal aorta and placed in a plastic tube containing 3.8% sodium citrate (citrate/blood: 1/9, v/v), and then plasma was separated through centrifugation of 3,000 rpm at 5°C for 15 min. Thromboxane B_2_ (TXB_2_) and 6-keto-PGF_1*α*_ were tested by Elisa kits (Enzo Life Sciences Inc., New York, USA), and the levels of vWF and TM were detected by the kits (Nanjing Jian Cheng Bioengineering Institute, Nanjing, China) according to instructions. All data were carried out by Varioskan Multifunctional full wavelength microplate reader (Thermo Fisher Scientific, USA) according to the manufacturers.

### 2.10. Quantitative Real-Time PCR Procedures

Total RNA was extracted from rat thoracic aorta according Trizol kit and treated with DNase I. The reverse transcription of the cDNA was carried out by a reverse transcription kit. The above operations are carried out according to the instructions. RNA and cDNA were stored at −70°C. PCR amplification reaction system is 20 *μ*L (SYB green 10 *μ*L, Primer 1 *μ*L, c DNA 1 *μ*L, DNase-free 8 *μ*L), 95°C × 3 min predegeneration, 95°C × 5 s degeneration, 56°C × 1 min annealing, 40 cycles. Primer sequences are designed and synthesized by Nanjing Jin Rui Biological Technology Co., Ltd. (Table 1). The results were analyzed by relative quantitative method, and the glyceraldehyde-3-phosphate dehydrogenase (GAPDH) mRNA was used as internal reference.

### 2.11. Date Analysis

All results were presented with the mean values ± SD. The data were analyzed by one-way analysis of variance (ANOVA) followed by the LSD test for multiple comparisons with the SPSS package (Version 22.0, SPSS Inc., US). A value of *P* < 0.05 were considered statistically significant.

## 3. Results

### 3.1. Standardization of TFE

**Figure 1:**
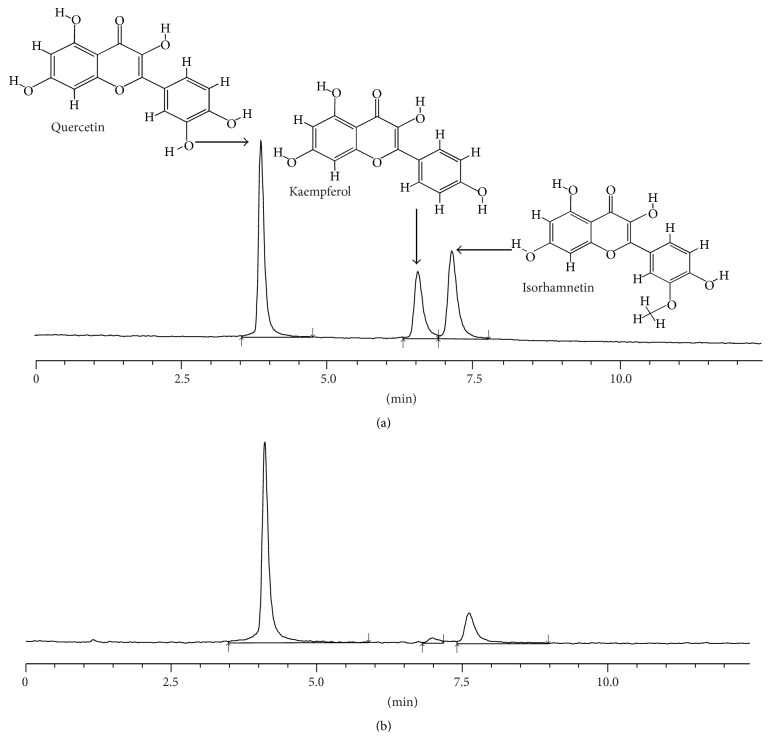
Typical high-performance liquid chromatography profile of three known standards (a) and total flavonoid aglycones obtained by hydrolyzing (b) at an absorbance of 370 nm.

A typical HPLC fingerprint of TFE was shown in Figure 1. Three major components were determined by a HPLC method. They were well separated and the retention times are 4.0, 6.8, and 7.4 min for quercetin, kaempferol, and isorhamnetin, respectively. The components of TFE were quantified by comparing the peak area with three known standards. As a result, the total flavonoid aglycones content was 30.94%. TFE contained quercetin (22.51%), kaempferol (0.95%), and isorhamnetin (7.48%).

### 3.2. Effect of TFE on WBV

**Figure 2:**
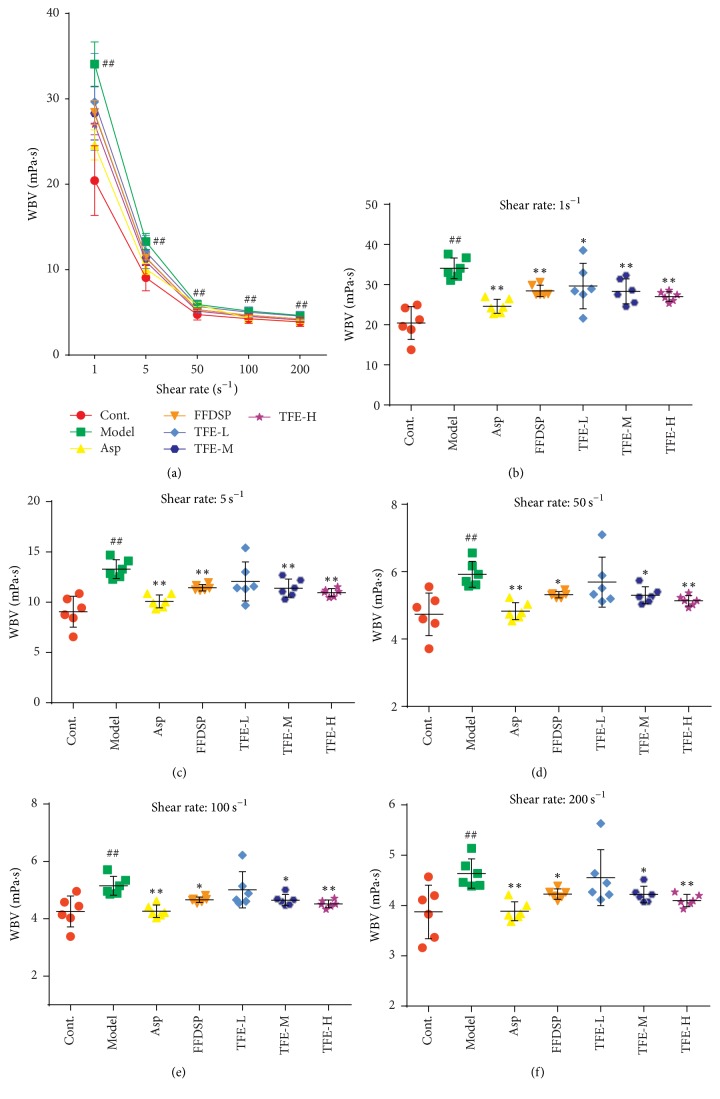
Effects of TFE on whole blood viscosity (WBV) at various shear rates. *n* = 6, ^##^*P* < 0.01 compared with control group. ^*∗*^*P* < 0.05, ^*∗∗*^*P* < 0.01 compared with model group. Highly significant change (*P* < 0.01) was recorded at all shear (ranging from 1 to 200 s^−1^), rates between control and model group (a). WBV at 1, 5, 50, 100, and 200 s^−1^ rates (b–f).

The effect of TFE on the WBV is shown in Figure 2. WBV was increased at 1 s^−1^, 5 s^−1^, 50 s^−1^, 100 s^−1^, and 200 s^−1^ shear rates in the model group in contrast to the control group with statistical significance (*P* < 0.01). The WBV was decreased at all shear rates in the THF-H as well as THF-M groups compared to the model group (*P* < 0.05 or 0.01). And WBV decreased at 1 s^−1^ shear rate in the THF-L group compared to the model group (*P* < 0.05). The positive control, aspirin, and FFDSP significantly decreased the WBV in comparison with the model group (*P* < 0.05 or 0.01). TFE concentration groups significantly inhibited the increase of WBV caused by adrenaline plus ice-water bath, but the effect was not significant in the experimental concentration range. There was no significant difference in WBV among TFE-L, TFE-M, and TFE-H groups.

### 3.3. Effect of TFE on Histopathology

**Figure 3:**
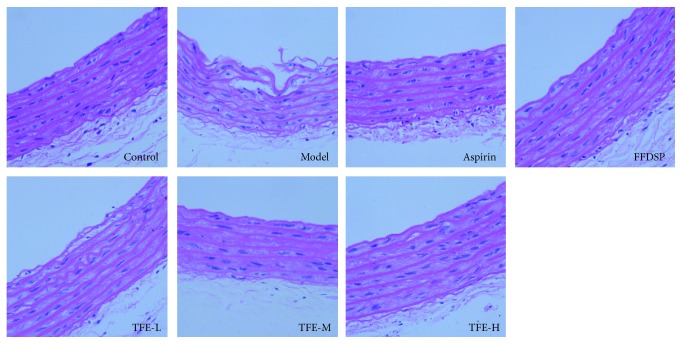
Effect of TFE in pathological photomicrograph of rats aorta: pathological photomicrograph of rats stained by hematoxylin and eosin (H.E. ×400).

Pathologic observation of vascular endothelial injury was manifested in Figure 3. In the normal group, the tunica intima of the aorta was smooth, and the smooth muscle cells of the middle membrane were neatly arranged and no smooth muscle cell proliferation. In the model group, aortic endothelium of the tunica intima was dislocated or even broken, and the elastic fibers were proliferated and smooth muscle cells arranged irregularly. In the ASP, TFE-M, and TFE-H groups, the endothelial cells had a lighter degree of shedding, and the inner cortex was clearer and more complete.

### 3.4. Effect of TFE on PT, APTT, and FIB

**Figure 4:**
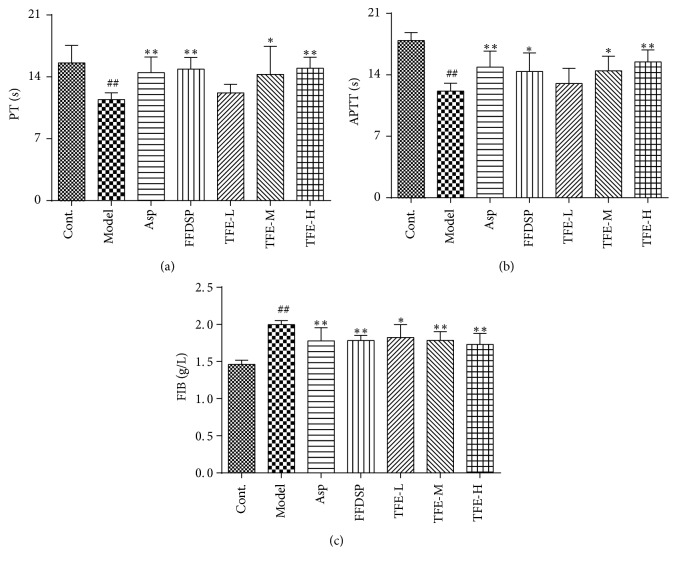
Effect of TFE on PT (a), APTT (b), and FIB (c). *n* = 6, ^##^*P* < 0.01 compared with control group. ^*∗*^*P* < 0.05, ^*∗∗*^*P* < 0.01 compared with model group.

As shown in Figure 4, PT and APTT were decreased, and level of FIB was downregulated effectively in model group rats compared with the control group (*P* < 0.01). The therapy of TFE at the dosage of 0.24 or 0.48 g/kg was able to lengthen PT and APTT (*P* < 0.05 or 0.01) and decrease FIB content significantly (*P* < 0.01). With the increase of the concentration, effects of TFE on the decrease of PT and APTT and the increase of FIB content in the model rats were gradually enhanced, and the difference between THE-L and THE-H group was significant (*P* < 0.01). However, there was no significant dose-effect relationship among TFE-L, TFE-M, and TFE-H groups. The effects of THE-M and THE-H on the decrease of PT and APTT and the increase of FIB content were similar to those of Asp and FFDSP groups.

### 3.5. Determination of TXB_2_ and 6-Keto-PGF_1*α*_

To evaluate the secretion of vascular endothelium, the plasma content of TXB_2_ and 6-keto-PGF_1*α*_ in rats was detected. As shown in Table 2, compared with the blank group, the contents of A and B in the model group were significantly different. The therapy with TFE at the dosage of 0.48 g/kg could effectively counterbalance the increase of TXB_2_ level. Treatment with TFE at the dosages of 0.24 and 0.48 g/kg could significantly decrease level of 6-keto-PGF_1*α*_. However, the dose-effect relationship of the TFE-L, TFE-M, and TFE-H groups in the experimental concentration range was not obvious. Meanwhile, treatment of TFE decreased the rate of TXB_2_/6-keto-PGF_1*α*_ compared with that in the model group (*P* < 0.01). THE-M group showed the strongest effect, and there was significant difference between TFE-L and TFE-M groups (*P* < 0.05). But there was no dose-effect relationship among TFE-L, TFE-M, and TFE-H groups.

### 3.6. Determination of vWF and TM

**Figure 5:**
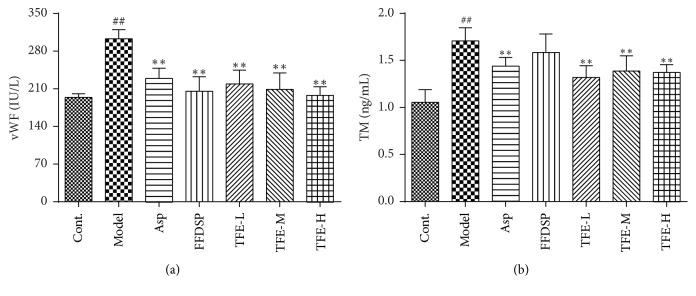
Determination of vWF (a) and TM (b) in the plasma. *n* = 6, ^##^*P* < 0.01 compared with control group; ^*∗∗*^*P* < 0.01 compared with model group.

As shown in Figure 5, the plasma vWF level in blood stasis model rats (303.32 ± 16.85 IU/L) was prominently higher than that of blank control group (193.99 ± 6.92 IU/L) (*P* < 0.01), whereas the plasma TM level in model rats (1.71 ± 0.14 ng/mL) was markedly lower than that of blank control group (1.05 ± 0.13 ng/mL) (*P* < 0.01). Compared with model rats, TFE can significantly downregulate vWF level and TM level in plasma (*P* < 0.01). The plasma vWF level of TFE-L, TFE-M, and TFE-L groups was 219.29 ± 25.59, 209.05 ± 30.58, and 197.78 ± 16.27 IU/L, respectively. The plasma TM level of TFH-L, TFE-M, and TFE-L groups was 1.32 ± 0.17, 1.39 ± 0.16, and 1.37 ± 0.09 ng/ml, respectively. Thus, with the increase of TFE concentration, plasma vWF content decreased gradually and plasma TM content also increased trend, but the two in the experimental concentration range of dose-effect relationshipare not significant.

### 3.7. Analysis of Quantitative Real-Time PCR

**Figure 6:**
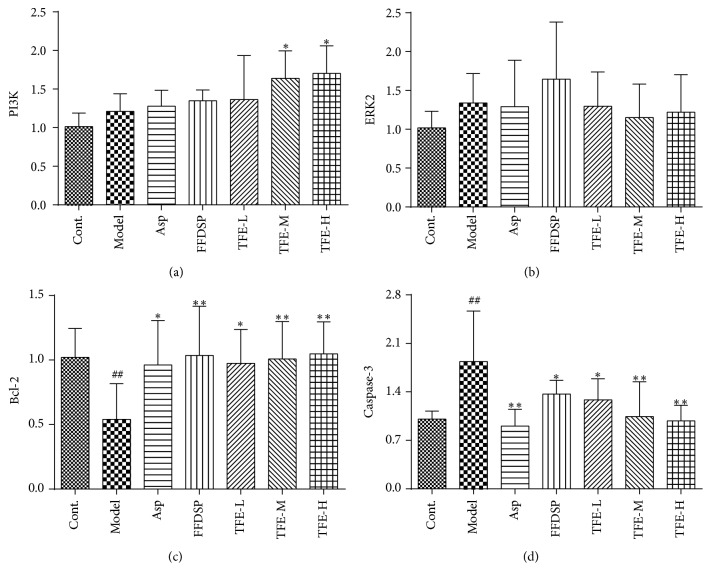
The mRNA expressions of PI3K (a), Erk2 (b), Bcl-2 (c), and caspase-3 (d). *n* = 6, ^##^*P* < 0.01 compared with control group. *n* = 6, ^*∗*^*P* < 0.05, ^*∗∗*^*P* < 0.01 compared with model group.

The magnitude and direction of changes in expression of the four genes affected by TFE pretreatment were confirmed by qPCR (Figure 6). Expressions of PI3K and ERK2 had an upregulation with no significant increase (*P* > 0.05) in the blood stasis model. The expression of Bcl-2 was significantly decreased (*P* < 0.05) and the expression of caspase-3 was significantly increased (*P* < 0.01) in the blood stasis model. Moreover, there were no visible differences in ERK2 in each administration group (*P* > 0.05). Compared with the model group, the expressions of PI3K and Bcl-2 were significantly increased (*P* < 0.05 or 0.01) and the expressions of caspase-3 decreased (*P* < 0.05 or 0.01) by TFE pretreatment. Expressions of PI3K and Bcl-2 in TFE-L, TFE-M, and TFE-L groups were similar and were close to those of the positive drug groups. The expression of caspase-3 in Asp, FFDSP, and TFE groups was significantly decreased (*P* < 0.05 or 0.01), and effect of THE-H group was similar to that of Asp group. With the increase of TFE concentration, expressions of PI3K mRNA and Bcl-2 mRNA increased, and the expression of caspase-3 mRNA decreased, but there was no significant dose-effect relationship in TFE-L, TFE-M, and TFE-L groups.

## 4. Discussion

In this study, we believe that TFE has a definite protective effect against vascular endothelial injury in blood stasis model rats. For one thing, we found that TFE could relieve WBV in with blood stasis rats (Figure 2). For another thing, further experimental results investigated that TFE could increase PT and APTT, which indicated that the beneficial effect of TFE on plasma viscosity might be partly related to the intrinsic and extrinsic coagulation system [16, 17]. As shown in Figure 3, pretreatment with TFE in blood stasis rats was able to prevent the increase in FIB, which suggested that the decrease in TFE to whole blood viscosity was mediated, at least partly, by the inhibition of an increase in FIB [18]. Meanwhile, histological examination results indicate that TFE also played a very important protective role in prevention of vascular endothelial injury (Figure 4).

In our experiments, rat models of blood stasis were established by a high dose of adrenaline with ice-water bath. Due to the dual effects of a large number of exogenous adrenaline injected and the secretion of a large amount of endogenous adrenaline under ice-water bath stimulation, the chemical stimulation of formaldehyde, hydrogen peroxide, and ammonia produced by adrenaline metabolism and the vasoconstrictive stress of its biological effects could lead to this peripheral vascular injury in model rats [19]. Large doses of adrenaline could cause strong contraction of blood vessels. This could cause blood pressure and blood shear stress changes, resulting in mechanical damage of the vascular wall [20, 21]. Meanwhile, the stimulation of biomechanics can cause the endothelial cells to produce various kinds of active substances, resulting in the increase of permeability and abnormalities of form and quantity [22, 23]. Therefore, adrenaline excessive deamination is considered to be a high risk factor for cardiocerebrovascular disease [24].

It is generally known that the vascular endothelial cells play a crucial role in reducing vascular permeability, antithrombotic, and regulating vascular smooth muscle function. The endothelial cells ingest AA (arachidonic acid) in circulating blood and produce TXA_2_ or PGI_2_, respectively, through the metabolic pathway of epoxy synthase. They form a very precise regulation mechanism in vivo. This mechanism is an important part of protecting endothelial cells from injury and plays an important role in regulating platelet adhesion aggregation, vascular tension, and thrombosis and in [25, 26]. Since TXA_2_ and PGI_2_ are extremely unstable, the contents of the stable metabolites TXB2 and 6-keto-PGF1*α* of TXA_2_ and PGI_2_ are often measured to represent the contents of A and B. Our study results suggested that TFE at the dosage of 0.48 g/kg might significantly downregulate serum TXB_2_ level and upregulate serum 6-keto-PGF_1*α*_ level in blood stasis rat, accompanied with the T/K decrease (Table 2, *P* < 0.01 compared with model group), indicating that the antithrombotic action of TFE was associated with the regulation of TXA_2_ and PGI_2_.

Furthermore, if the morphological structure of vascular endothelial cells is damaged or change in function will hinder the normal operation of blood, some landmarks' level of vascular endothelial injury in the blood will rise, such as vWF and TM. VWF is a macromolecular glycoprotein synthesized by vascular endothelial cells. Under normal circumstances, endothelial cells can secrete a small amount of vWF [27]. However, under the condition of vascular injury or some pathological conditions, endothelial cells stimulate and VWF release increase [28]. TM is a complete membrane glycoprotein containing 575 of amino acids and 5 major groups and is present in the endothelial cell membrane surface of arteries, veins, capillaries, and lymphatics [29]. When the blood vessels are damaged, the protein on the surface of the endothelial cell is hydrolyzed or shedding into the blood, resulting in elevated levels of TM in the plasma [30]. TFE administration could significantly decrease vWF level and TM level (Figure 5). These results indicated that TFE could attenuate vascular endothelial injury and might regulate the thrombogenesis by downregulating vWF level and TM level.

Studies have pointed out that the PI3K/ERK1/2 activation is vital in order to exert effects on cell proliferation and regulate vessel remodeling [31, 32]. Bcl-2 will act by regulating the release from mitochondria of caspases activators [33]. Caspases are essential proteins in cell apoptosis; in particular, caspase-3 is the crucial effector and a focal point of the apoptosis pathway. The apoptosis of human cerebrovascular endothelial cells (HCMECs) induced by hypoxia/reoxygenation was associated with decreased expression levels of Bcl-2 and increased expression levels of Bax and activated caspase-3 [34]. Cheng et al. reported that treatment with 100–400 *μ*g/ml THF could significantly attenuate the H_2_O_2_-induced VECs apoptosis by downregulating the caspase-3 expression respectively [10]. In the current study, it was observed that TFE protected VECs from blood stasis model rats with vascular endothelial injury by upregulating PI3K and Bcl-2 expression levels and downregulating caspase-3 expression levels (Figure 6). Additionally, it was demonstrated that VECs protection by TFE was concentration-dependent. According to results, we confirm that vascular endothelial injury could be restored by TFE pretreatment in the rat model through activating the PI3K/Bcl-2/caspase-3 pathway associated with VECs apoptosis development. However, effects of TFE pretreatment on mitochondrial control of apoptosis remain elusive, and in-depth studies are needed to confirm these additional mechanisms.

## 5. Conclusions

In conclusion, the results of this study support the concept that TFE can regulate the balance of TXA_2_/PGI_2_ and improve the function of vascular endothelial cells effectively and regulate the thrombogenesis by downregulating levels of vWF and TM. We tentatively put forward that TFE protects vascular endothelial in blood stasis model rats by increasing the expression of PI3K and Bcl-2 and reducing the expression of caspase-3. TFE has great therapeutic potential to attenuate blood stasis-related vascular endothelial injury.

## Figures and Tables

**Table 1:** Primers list for real-time PCR analysis.

Gene	Primer	Oligonucleotide sequence (5′~3′)	Product length
PI3K	F	GTGTAAAACCGTCGTAAG	121
	R	AACAGCAAAACATAATCG	
Erk-2	F	AGCATTACCTTGACCAGC	137
	R	GTTCCACGGCACCTTAT	
Bcl-2	F	GGGAGAACAGGGTATGA	145
	R	GCTGGAAGGAGAAGATG	
Caspase-3	F	GGACTGCGGTATTGAGA	99
	R	GGTGCGGTAGAGTAAGC	
GAPDH	F	CAAGTTCAACGGCACAG	138
	R	CCAGTAGACTCCACGACAT	

**Table 2:** Effect on TXB_2_ and 6-keto-PGF_1*α*_ in plasma (mean ± SD, *n* = 6, ^#^*P* < 0.05, ^##^*P* < 0.01 compared with control group; ^*∗*^*P* < 0.05  ^*∗∗*^*P* < 0.01 compared with model group).

Group	*n*	Dose/g·kg^−1^	pg/ml		TXB_2_/6-keto-PGF_1*α*_
			TXB_2_	6-Keto-PGF_1*α*_	
Control	6	—	153.82 ± 40.86	31448 ± 5441	0.0056 ± 0.0017
Model	6	—	283.37 ± 90.07^#^	13694 ± 3497^##^	0.028 ± 0.012^##^
Aspirin	6	0.03	106.28 ± 21.48^*∗∗*^	6645 ± 2277^*∗∗*^	0.0168 ± 0.0038^*∗∗*^
FFDSP	6	0.45	367.88 ± 115.17	31504 ± 3432^*∗∗*^	0.0117 ± 0.0039^*∗∗*^
TFE-L	6	0.12	274.82 ± 130.03	16927 ± 3489	0.0171 ± 0.0096^*∗∗*^
TFE-M	6	0.24	205.12 ± 68.04	29532 ± 5228^*∗∗*^	0.0074 ± 0.0036^*∗∗*^
TFE-H	6	0.48	186.04 ± 55.86^*∗*^	24739 ± 3991^*∗∗*^	0.008 ± 0.0034^*∗∗*^
